# A *Bacillus* Flagellar Motor That Can Use Both Na^+^ and K^+^ as a Coupling Ion Is Converted by a Single Mutation to Use Only Na^+^

**DOI:** 10.1371/journal.pone.0046248

**Published:** 2012-09-25

**Authors:** Naoya Terahara, Motohiko Sano, Masahiro Ito

**Affiliations:** 1 Graduate School of Frontier Biosciences, Osaka University, Suita, Osaka, Japan; 2 Graduate School of Life Sciences, Toyo University, Oura-gun, Japan; 3 Bio-Nano Electronics Research Centre, Toyo University, Kawagoe Saitama, Japan; University of Groningen, Netherlands

## Abstract

In bacteria, the sodium ion (Na^+^) cycle plays a critical role in negotiating the challenges of an extremely alkaline and sodium-rich environment. Alkaliphilic bacteria that grow optimally at high pH values use Na^+^ for solute uptake and flagellar rotation because the proton (H^+^) motive force is insufficient for use at extremely alkaline pH. Only three types of electrically driven rotary motors exist in nature: the F-type ATPase, the V-type ATPase, and the bacterial flagellar motor. Until now, only H^+^ and Na^+^ have been reported as coupling ions for these motors. Here, we report that the alkaliphilic bacterium *Bacillus alcalophilus* Vedder 1934 can grow not only under a Na^+^-rich and potassium ion (K^+^)-poor condition but also under the opposite condition in an extremely alkaline environment. In this organism, swimming performance depends on concentrations of Na^+^, K^+^ or Rb^+^. In the absence of Na^+^, swimming behavior is clearly K^+^- dependent. This pattern was confirmed in swimming assays of stator-less *Bacillus subtilis* and *Escherichia coli* mutants expressing MotPS from *B. alcalophilus* (BA-MotPS). Furthermore, a single mutation in BA-MotS was identified that converted the naturally bi-functional BA-MotPS to stators that cannot use K^+^ or Rb^+^. This is the first report that describes a flagellar motor that can use K^+^ and Rb^+^ as coupling ions. The finding will affect the understanding of the operating principles of flagellar motors and the molecular mechanisms of ion selectivity, the field of the evolution of environmental changes and stresses, and areas of nanotechnology.

## Introduction

Bacterial flagella act as rigid propellers for cell locomotion in liquid and on surfaces, and their rotation is powered by electrochemical gradients of either protons (H^+^) or sodium ions (Na^+^) across the bacterial cytoplasmic membrane [1], [2]. The flagellar structure is composed of a helical filament, a basal body that is embedded in the cytoplasmic membrane, and a hook that connects the filament to the basal body. The motor is divided into two parts: the rotor and the stator. The stators are most often called Mot complexes which function as ion channels and act as the energy conversion unit which rotates a flagellar rotor when coupling ions pass through the Mot complexes. Mot complexes have been shown to contain four MotA-like proteins, each of which contains four transmembrane segments (TMS), and two MotB-like proteins, each of which contains a single TMS [1]–[3]. The MotB-like proteins have a major role in determining ion-coupling specificity [4], [5]. In most bacteria H^+^-coupled motility is widely documented. However, the work of Imae and colleagues in the 1980s revealed that extremely alkaliphilic *Bacillus* species use only Na^+^ gradients for motility [6], [7]. Evidence from an increasing number of bacteria indicates that they utilise dual flagellar systems that have different ion specificities that contribute to swimming under particular physical-chemical conditions, such as pH, salinity and viscosity [8]–[12]. Therefore, although extremely alkaliphilic *Bacillus* species contain only sodium-coupled MotPS and *Escherichia coli* contains only proton-coupled MotAB, salt-tolerant *Vibrio alginolyticus* possesses proton-coupled MotAB and sodium-coupled PomAB, and *Bacillus subtilis* possesses proton-coupled MotAB and sodium-coupled MotPS [13]. In 2008, we reported that alkaliphilic *Bacillus clausii* KSM-K16 utilises a novel form of dual-ion coupling for motility, in which a single MotAB stator uses both sodium and protons at different pH ranges [14]. Deductions from studies of alignments of the MotB and MotS subunits of sodium- and proton-coupled motility systems facilitated conversion of single-ion coupled stators to dual use and the *B. clausii* dual-ion stator to single-ion use of either sodium or protons [14]. Recently, Schlegel et al reported that ATP synthesis in *Methanosarcina acetivorans* can be driven by the H^+^ or Na^+^ gradients [15]. No stators that can use potassium ions, which is reported here for the MotPS system of alkaliphilic *Bacillus alcalophilus* Vedder 1934, have previously been described. This strain, which was isolated from human feces in 1934, is a classic alkaliphilic strain because it was among the very first alkaliphilic “extremophiles” to be described in the literature [16].

The *Bacillus alcalophilus* Vedder 1934 genome sequence was not yet accessible at the start of this project (BioProject accession number PRJNA13375). Therefore, the uncharacterised flagellar stator genes from this strain were amplified by PCR using primers that were based on the conserved sequences among the *motAB* and *motPS* genes of *Bacillus* species. The sequencing of each PCR product revealed that a single set of genes encoded a MotPS-like pair of proteins (GenBank accession JN561694). Subsequently, one of us (MI) participated in a *B. alcalophilus* Vedder 1934 genome sequencing project demonstrated that, in fact, only a single mot system is present (This Whole Genome Shotgun project has been deposited at DDBJ/EMBL/GenBank under the accession ALPT00000000. The version described in this paper is the first version, ALPT01000000). We use the designation “BA-MotPS” for this Mot stator. The BA-MotP and MotS proteins were closely related to the *B. subtilis* MotP and MotS proteins that constitute the sodium-coupled *B. subtilis* stator complex (49% and 48% identity and 75% and 68% similarity, respectively). They less closely resembled the *B. subtilis* MotA and MotB proteins that constitute a proton-coupled *Bacillus subtilis* stator complex (39% and 29% identity and 61% and 55% similarity, respectively). The swimming behaviour of *B. alcalophilus* Vedder 1934 was also studied under several conditions. Since this strain is not genetically accessible, we used stator-less mutant strains of *B. subtilis* (Δ*motAB* Δ*motPS*) and *Escherichia coli* RP6894 (Δ*motAB*) and a potassium uptake-deficient (Δ(*kdpABC*) *trk*Δ1 Δ*trkA*) mutant strain of *E. coli* TK2420 as the host for experiments in which the functional properties of BA-MotPS were studied. We were also able to identify mutational changes in BA-MotS that converted the bi-functional BA-MotPS stator to one that cannot use K^+^ or Rb^+^ when the sodium concentration is very low as the native BA-MotPS can.

## Materials and Methods

### Bacterial strains and growth conditions

The strains and plasmids used in this study are shown in Table 1. *Bacillus alcalophilus* Vedder 1934 cells and *Bacillus pseudofirmus* OF4 811MM were grown aerobically at 30°C in malate–yeast extract medium (MYE) at pH 10.5 [17] or KMYE medium. MYE medium contained 50 mM Na_2_CO_3_, 1 mM K_2_HPO_4_, 0.1 mM MgSO_4_, 0.01%(w/v) yeast extract, 2.5 mM sodium malate (pH 9.0), and 1%(v/v) trace elements per liter of deionized water (pH 10.5). A similar medium containing 50 mM Na_2_HPO_4_ instead of Na_2_CO_3_ was occasionally used as MYE medium (pH 7.5), and 50 mM K_2_CO_3_ instead of Na_2_CO_3_ was occasionally used as KMYE medium (pH 10.5), and KMYE medium containing 50 mM K_2_HPO_4_ was occasionally used instead of K_2_CO_3_ as KMYE medium (pH 7.5). For assays of swimming speed in liquid, 30 mM Tris-HCl (pH 9.0) containing 5 mM glucose plus the indicated amounts of sodium, potassium or rubidium were used at the indicated values of pH and added EIPA (5-(*N*-ethyl-*N*-isopropyl)-amiloride). The actual final Na^+^ and K^+^ concentration of each assay medium was determined with a flame photometer (Model AMA-175, Tokyo Koden, Tokyo, Japan) calibrated with standard Na^+^ solutions of known concentrations. The effects of increasing concentrations of KCl on the growth of *E. coli* TK2420 were determined as described previously [18] in a defined medium [19]. *Bacillus subtilis* 168 strain BR151MA (wild type), its derivatives and *E. coli* strain RP6894 were grown at 37°C in LB medium. *E. coli* strain TK2420 was grown at 37°C in LBK medium. When necessary, a particular medium was supplemented with erythromycin (0.3 µg/ml), neomycin (7.5 µg/ml), chloramphenicol (5 µg/ml) or ampicillin (100 µg/ml). For assays of swimming speed in liquid, 10 mM phosphate buffer that contained 5 mM glucose and the indicated amounts of sodium and potassium was used at the indicated pH values. For growth assays of *E. coli* strain TK2420 cells, TK2420 medium (pH 7.0) consisted of 33.6 mM Na_2_HPO_4_, 20.3 mM NaH_2_PO_4_, 1.1 mM citric acid, 7.6 mM (NH_4_)_2_SO_4_, 6 µM FeSO_4_, 830 µM MgSO_4_, 10 mM glucose, 1 µg/ml thiamine, 100 µg/ml ampicillin and 0.06% arabinose was used with the indicated amounts of potassium. The cells were grown at 37°C with shaking, and their growth was monitored by measuring the absorbance at 600 nm.

**Table 1:** Bacterial strains and plasmids used in this study.

Strain or plasmid	Description	Source or reference
*E. coli* Strain		
DH5αMCR	F^−^ *mcrA*Δ*1* (*mrr-hsd RMS-mcrBC*) Φ80*dlacZ* Δ(*lacZYAargF*) *U169 deoR recA1 endA1 supE44 λthi-1 gyr-496 relA1*	Stratagene
TK2420	F^−^ *thi rha lacZ nagA* Δ(*kdpFAB*) Δ(*trk-mscL*) *trkD1*	[49]
RP6894	Δ*motAB*	[48]
*Bacillus subtilis* strains		
BR151MA	*lys3 trpC2* (wild type)	[50]
ΔABΔPS	*lys3 trpC2* Δ*motAB*::*ery* Δ*motPS*::*neo*	[13]
BA-PS	ΔABΔPS *amyE*::P*_motAB_*-*motAB* from Vedder 1934	This study
BA-PS-MotS_M33L	BA-PS introduced a mutation in MotS_M33L	This study
*Bacillus alcalophilus* strain		
Vedder 1934	wild type, ( = JCM5652)	[16]
*Bacillus pseudofirmus* OF4 strains		
811M	Met^−^ (wild type)	[51]
811MM	Met^−^ up-motile variant	[45], [52]
Plasmids		
pDR67	*amyE* integration vector with Cm^r^ gene and P_spac_ promoter upstream of multiple cloning site	[53]
pBAD24	Expression vector containing P_BAD_ promoter	[54]
pDR-PS	pDR67+P*_motAB_*-*motPS* from Vedder 1934	This study
pDR-PS-MotS_M33L	pDR-PS introduced a mutation in MotS_M33L	This study
pBAPS	pBAD24+*motPS* from Vedder 1934	This study
pBAPS-MotS_M33L	pBAPS introduced a mutation in MotS_M33L	This study

### Construction of plasmid coding *B. alcalophilus motPS* under *B. subtilis P_motAB_* promoter and its point mutations

For cloning of the *B. alcalophilus motPS* gene under the *B. subtilis P_motAB_* promoter, we performed gene splicing by overlap extension (gene SOEing) method based on the PCR method. The primers were synthesized based on sequences from *B. subtilis* and *B. alcalophilus* and are listed in Table 2. The *B. subtilis P_motAB_* promoter was amplified by PCR using *B. subtilis* chromosomal DNA as the template with BSAB-BamHI-F primers designed for a BamHI site, and BSpAB-BAPS-R primers introduced a sequence of the N-terminal region of the *B. alcalophilus motPS* gene. The *B. alcalophilus motPS* gene was amplified by PCR using *B. alcalophilus* chromosomal DNA as the template with BSpAB-BAPS-F primers introducing a sequence of the *B. subtilis P_motAB_* promoter and BAPS-SphI-R. These two products share an overlap region. We performed PCR with these two products as the template to join the *B. subtilis P_motAB_* promoter and the *B. alcalophilus motPS* gene. The amplified fragment was digested by BamHI and SphI and cloned into BamHI and SphI-digested pDR67, yielding pDRPS. To introduce an amino acid substitution into *the B. alcalophilus motS* gene, we used the gene SOEing method. We synthesized pairs of mutant primers to the sense strand of the *B. alcalophilus motS* gene, with a mismatch at the mutation site. We amplified the *B. alcalophilus motPS* under control of a *B. subtilis P_motAB_* promoter by PCR with mutant primers, and cloned it into BamHI and SphI-digested pDR67, yielding pDRPS -MotS_M33L. The presence of the *motS* mutation was confirmed by DNA sequencing. Each plasmid was used to transform particular mutants to a chloramphenicol-resistance, amylase-negative phenotype for the pDR67 derivative. Recombinant transformants were selected by conventional techniques, and the presence of the insert was confirmed.

**Table 2:** Oligonucleotides used in this study.

Primer	Sequence	Accession number and corresponding sequence (nt)
BSAB-BamHI-F	5′-cgcggaTCCGCGACCTCTTGCTTCCGACA-3′	Z99111 (23924–23902 (minus strand))
BSpAB-BAPS-F	5′-AAAAAAGGATTTGGTGAAAACTATGAAAAAGCTAGATCTGATGA-3′	Z99111 (23633–23609 (minus strand))
		JN561694 (1458–1480)
BSpAB-BAPS-R	5′-TCATCAGATCTAGCTTTTTCATAGTTTTCACCAAATCCTTTTTT-3′	JN561694 (1480–1458 (minus strand))
		Z99111 (23609–23633)
BAPS-SphI-R	5′-acAtgcAtgCCCAAGGAATATTGTCTCCTCG-3′	JN561694 (3020–2999 (minus strand))
PS(BA)-S-M33L-F	5′-ATGACGTTGATTTTAGTGTTTTTTGTTcTGTTATTTTCGATGTCAGAAAT-3′	JN561694 (2326–2375)
PS(BA)-S-M33L-R	5′-AAAAAACACTAAAATCAACGTCAT-3′	JN561694 (2349–2326 (minus strand))
BAPS-NcoI-F	5′-catGccatggATGAAAAAGCTAGATCTGATGACA-3′	JN561694 (1458–1481)
BAPS-PstI-R	5′-aaaaCtGcagTTAATAAGTATCATCCTCTTCATA-3′	JN561694 (2994–2971 (minus strand))

Extra nucleotides that were added to introduce restriction sites are underlined.

Substituted nucleotides that were added to introduce point mutation sites are shown by a small letter.

For cloning of the *B. alcalophilus motPS* genes under an arabinose *P_BAD_* promoter of the pBAD24 plasmid, the primers digested by NcoI and PstI were synthesized based on sequences from *B. alcalophilus*. The *B. alcalophilus motPS* genes were amplified by PCR using pDRPS and pDRPS-MotS_M33L as the template. These amplified fragments were cloned into NcoI and PstI-digested pBAD24, yielding pBAPS and pBAPS -MotS_M33L.

### Measurement of intracellular potassium and sodium concentration

*E. coli* TK2420 cells were grown at 37°C or 10 hour in TK2420 medium contained 25 mM or 50 mM KCl. The cells were harvested by centrifugation (3,000×g, 10 min, 25°C) and washed by suspension in the same medium. Then, the cells were resuspended in 5 ml of 300 mM sucrose solution, and the cell protein was measured by the Lowry method using 100 µl of the cell suspension. The rest of the suspension was harvested and resuspended in 5 ml of 5% trichloroacetic acid (TCA) solution. After being boiled for 10 min, cell debris was removed by centrifugation and potassium and sodium were measured by atomic absorption spectrometry (Model Z-8000, Hitachi, Japan) that was calibrated with standard potassium and sodium solutions of known concentrations. The intracellular concentration was calculated with an assumed cell volume of 3 µl/mg cell protein [20], [21].

### Measurement of swimming speed

For measurement of swimming speed, *B. alcalophilus* Vedder 1934 cells were grown aerobically on KMYE medium (pH 10.5) overnight at 30°C. The culture was then inoculated into 20 ml of a fresh KMYE medium (pH 10.5) at an A_600_ of 0.01, and grown aerobically at 30°C. Growth of *B. pseudofirmus* OF4 811MM cells used MYE medium under the same conditions. Highly motile cells in the late logarithmic phase were harvested by filtration on OMNIPORE membrane filters (0.45 µm) and washed 3-times with 2 ml of the indicated buffer for measurement of swimming speeds. Cells were suspended in 0.5 ml of the same buffer, and incubated at 30°C for 10 min. Cell motility was observed under a dark-field microscope using a Leica DMRE microscope and Progressive 3CCD color video camera system (Model DXC-9000, SONY, Tokyo, Japan) and recorded on videotape. Swimming speed was determined by direct tracing of the moving cells on the video monitor. The ionic strength of the assay buffers increased as the cation concentration was elevated. All results shown are the averages of three independent experiments in which the speed of 20 different cells was measured.

### Visualization of flagella

Flagella staining was carried out as described by Aono *et al.* [22]. *B. alcalophilus* cells were cultured at 30°C for 7 hours in KMYE medium or for 22 hours in MYE medium, and transferred gently to a microscope slide. The sample was air-dried and treated for 2 minutes with staining solution containing 5% (w/v) tannic acid, 0.75% (w/v) FeCl_2_, 0.01% NaOH, followed by ammoniac silver nitrate for 30 seconds. Observations of flagella were made using a Leica DMLB100 bright field microscope and Leica DC300F camera, Leica IM50 version 1.20 software (Leica Geosystems, Tokyo, Japan). The number of flagella/cell and flagellar length were measured for 55 cells by using ImageJ version 1.36b software (NIH, Bethesda, Maryland, USA).

### Western blot analysis

To investigate the effect of the mutation on the expression level of the stator protein in membrane fractions, three *B. subtilis* strains, ΔABΔPS, BA-PS and BA-PS-MotS-M33L, and six *E. coli* strains, RP6894 carrying pBAD24, pBAPS, or pBAPS-MotS-M33L, and TK2420 carrying pBAD24, pBAPS, or pBAPS-MotS-M33L, were grown as described above, harvested and washed in Tris Buffer (50 mM Tris–HCl pH 8.0). Cells were suspended in the same buffer and a protease inhibitor cocktail (SIGMA) was added. Membrane vesicles were prepared by breaking cells with a French Pressure cell [23], [24], except that the buffer used was 50 mM Tris-HCl, pH 8.0. 10 µg of membrane protein/µl from each sample was used for one-dimensional sodium dodecyl sulfate (SDS)-PAGE analyses. The same volume of SDS loading buffer was added to each sample, after which the proteins were separated on 10% polyacrylamide SDS gels (Bio-Rad). The gels were then transferred to nitrocellulose filters (Bio-Rad) electrophoretically by the application of 60 V for 3 h in Tris-glycine-methanol buffer (25 mM Tris, 192 mM glycine, 20% (v/v) methanol [pH 8.3]). The MotP protein from *B. alcalophilus* was detected by antibodies raised to synthetic peptides that corresponded to a conserved MotP region in *B. pseudofirmus* OF4 MotP residues 242–253, LEEKLSAFTREK. The corresponding sequence in *B. alcalophilus* Vedder 1934 is **L**Q**EKL**N**AF**N**R**Q**K**. An additional cysteine was added to the C terminus of the peptide. The peptides were conjugated to keyhole limpet hemocyanin and polyclonal antibodies were raised in rabbits (Operon Biotechnologies, Inc., Tokyo); a purified IgG fraction (Melon Gel IgG Spin Purification Kit, Thermo Fisher Scientific, USA) was used for the analyses. The protein concentration of the broken cell suspension was measured by the Lowry method with BSA as a standard. Goat anti-rabbit horseradish peroxidase (Bio-Rad) was also used as the second antibody for detection of anti-MotP antibody. ECL solution (Amersham Biosciences) was the usual detection reagent. A set of ECL Plus solution (Amersham Biosciences) and Can Get Signal immunoreactions enhancer solution (Toyobo) was used in some experiments, as indicated. A quantitative imaging system, Pluor-S MAX (Bio-Rad), was used for detection and analysis of chemiluminescence images.

## Results

### Multiple alignments of MotB-type stator proteins from *Bacillus* and *Vibrio* species and *B. alcalophilus*

**Figure 1:**
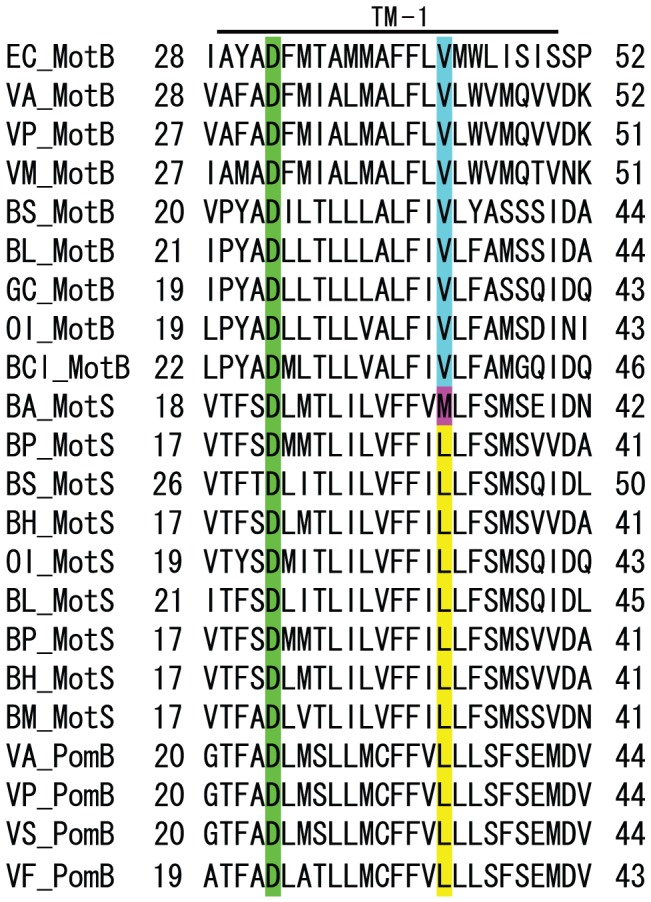
Stained flagellar of *B. alcalophilus* and alignment with flagella motor sequences from other bacteria. Alignments of the region containing the single transmembrane segment of *E. coli* MotB (EC_MotB), *B. subtilis* MotB (BS_MotB) and MotS (BS_MotS), *B. licheniformis* MotB (BL_MotB) and MotS (BL_MotS), *Geobacillus kaustophilus* MotB (GK_MotB), *Oceanobacillus iheyensis* MotB (OI_MotB) and MotS (OI_MotS), *B. clausii* MotB (BCl_MotB), *B. alcalophilus* MotS (BA_MotS), *B. pseudofirmus* MotS (BP_MotB), *B. halodurans* MotS (BH_MotB), *B. megaterium* MotB (BM_MotS), *V. alginolyticus* MotB (VA_MotB) and PomB (VA_PomB), *V. parahaemolyticus* MotB (VP_MotB) and PomB (VP_PomB), *V. mimicus* MotB (VM_MotB), *V. splendidus* PomB (VS_PomB), and *V. fisheri* PomB (VF_PomB). The position of D32 in EC_MotB is known to be critical for rotation and is highlighted in green. The MotAB of *B. clausii* can use Na^+^ instead of H^+^ to promote flagellar rotation at high pH values. The V37L mutation was critical for sodium selectivity and a combination of the V37L mutation and either the A40S or the G42S mutation was required for production of the BCl-MotB (the ninth line) form that exhibits sodium-coupling at low pH [14]. The position of V43 in EC_MotB (the first line) is conserved among all of the MotB-H^+^-type proteins and is highlighted in light blue. The position of L32 in BP_MotS (the eleventh line from the top) is conserved among all of the MotS-Na^+^-type proteins with the exception of BA_MotS and is highlighted in yellow. The same position in *B. alcalophilus* MotS encodes methionine instead of the conserved leucine residue, and it is highlighted with violet.

As illustrated in the alignment in Figure 1, proton-coupled MotB type stator subunits of numerous bacteria contain a conserved valine in the middle of the transmembrane segment. In contrast, the sodium coupled MotS and PomB type stator subunits contain a conserved leucine at the same location. Interestingly, the same site in BA-MotS contains a methionine. This led us to investigate the monovalent cation profiles for support of both growth and swimming of *B. alcalophilus*.

### Na^+^- or K^+^-dependent growth capacities of neutralophilic *B. subtilis*, alkaliphilic *B. pseudofirmus* OF4 and alkaliphilic *B. alcalophilus* at pH 7.5 and 10.5

**Figure 2:**
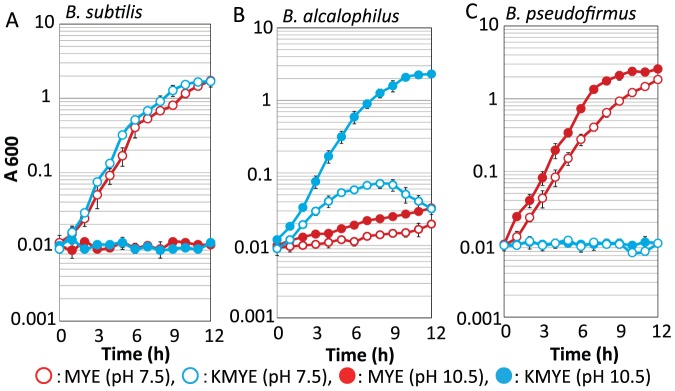
Growth of neutralophilic *B. subtilis* two alkaliphilic *Bacillus species*. *(A)*, *(B)* and *(C)* show the growth data for *B. subtilis*, *B. alcalophilus* and *B. pseudofirmus*, respectively. Growth in MYE medium (pH 7.5, red filled circle and pH 10.5, red open circle) and KMYE medium (pH 7.5, light-blue filled circle and pH 10.5, light-blue open circle) at 30°C was monitored at A600. The results are the averages of three independent experiments, with error bars representing the standard deviations.

We initially attempted to grow *B. alcalophilus* in MYE medium, pH 10.5, which is a typical malate-containing growth medium for alkaliphiles; we also used KMYE medium, which contains potassium instead of sodium. As measured using flame photometry, MYE medium is rich in sodium ions (189 mM) and poor in potassium ions (5.5 mM), and KMYE medium is rich in potassium ions (210 mM) and poor in sodium ions (0.7 mM). A similar medium that replaced the Na_2_CO_3_ and K_2_CO_3_ of the pH 10.5 media with the same molar concentrations of Na_2_HPO_4_·7H_2_O and K_2_HPO_4_ was used as the medium for experiments at pH 7.5. The cells of neutralophilic *Bacillus subtilis* (a control for growth at only neutral pH values) [25], alkaliphilic *B. pseudofirmus* OF4 (a control for completely Na^+^-dependent growth) [25] and *B. alcalophilus* were grown in MYE and KMYE medium at pH 7.5 and 10.5 (Figure 2B). While *B. subtilis* displays both Na^+^- and K^+^-dependent growth at pH 7.5, *B. pseudofirmus* OF4 displays only Na^+^-dependent growth at both pH values. Interestingly, *B. alcalophilus* could grow in all of the media tested, and the best growth was observed in KMYE medium at pH 10.5.

### *B. alcalophilus* Motility Responds to Added Sodium, Potassium and Rubidium

**Figure 3:**
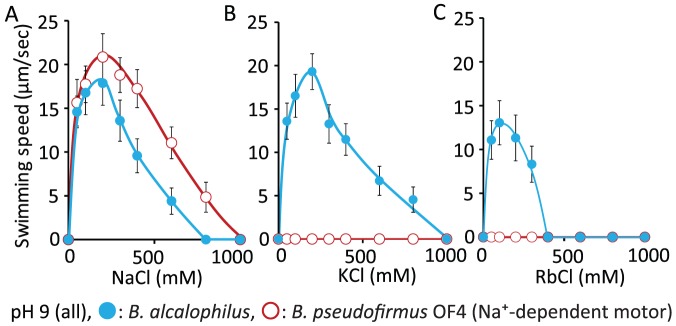
Motility of *B. pseudofirmus* and *B. alcalophilus* in liquid medium. *B. pseudofirmus* OF4 and *B. alcalophilus* cells in the logarithmic growth phase that were grown at 30°C in MYE medium (pH 10.5) and KMYE medium (pH 10.5), respectively, were harvested and resuspended in 30 mM Tris-HCl (pH 9.0) that contained 5 mM glucose and the indicated sodium *(A)*, potassium *(B)* or rubidium *(C)* concentrations as described in the Materials and Methods section. The red line and red open circles show the data for the *B. pseudofirmus* OF4 strain, and the blue line and blue filled circles show the data for the *B. alcalophilus* strain. The swimming speed was determined as described in the Materials and Methods section. The results that are shown represent the averages of three independent experiments in each of which the swimming speeds of 20 independent cells as calculated in each experiment. The error bars indicate the standard deviations of the values.

When grown at pH 10.5, cells of *B. alcalophilus* Vedder 1934 had an average of 3.0±1.2 and 3.0±1.4 flagella per cell, respectively, in MYE and KMYE medium in which Na^+^ was replaced with K^+^; the average flagellar length was 5.4±1.9 µm and 6.7±2.1 µm, respectively, in MYE and KMYE. The stained flagella of typical cell in KMYE is shown in Figure S1. To measure swimming speed, *B. alcalophilus* and *B. pseudofirmus* OF4 (a control for only Na^+^-dependent swimming) were grown aerobically on KMYE and MYE medium (pH 10.5) at 30°C, respectively. Swimming speed assays were then conducted in liquid buffer at pH 9.0 (Figure 3). The swimming speeds of *B. pseudofirmus* OF4 and *B. alcalophilus* both increased with increasing NaCl up to about 200 mM NaCl. Only swimming of *B. alcalophilus* speed was also stimulated by KCl or RbCl (Figure 3B, C), exhibiting robust enhancement by up to 200 mM KCl and enhancement of more modest swimming speeds up to 100 mM RbCl. No swimming was observed when *B. pseudofirmus* OF4 was tested in either KCl or RbCl assay buffer. These data suggest that the flagellar motor of *B. alcalophilus* can utilise K^+^, Rb^+^ and Na^+^ as coupling ions for the rotation.

**Figure 4:**
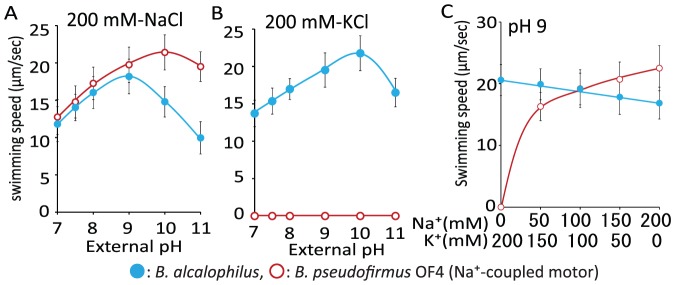
The swimming speed of two alkaliphiles dependent upon pH and concentrations of NaCl and KCl. The relationship between the swimming speed and several different pH values at 200 mM Na^+^ *(A)* or K^+^ *(B)* is illustrated. The relationship between swimming speed in 30 mM Tris-HCl containing 5 mM glucose (pH 9.0) and the various indicated concentrations of KCl and NaCl is shown in *(C)*. The red line and red open circles show the data for the *B. pseudofirmus* OF4 strain, and the blue line and blue filled circles show the data for the *B. alcalophilus* strain. The swimming speed was determined as described in the Materials and Methods section. The results that are shown represent the averages of three independent experiments in each of which the swimming speeds of 20 independent cells as calculated in each experiment. The error bars indicate the standard deviations of the values.

We next performed swimming speed assays in liquid buffer that contained 200 mM Na^+^ or 200 mM K^+^ at several different pH values to determine the pH that supported the fastest swimming speed for *B. pseudofirmus* OF4 and *B. alcalophilus* (Figure 4A, B). The maximal swimming speed of *B. pseudofirmus* OF4 in NaCl buffer occurred at pH 10 and the velocity was approximately 22 µm per second. The maximal swimming speed of *B. alcalophilus* in NaCl buffer was pH 9, and the velocity was approximately 18 µm per second. The maximum velocity of *B. alcalophilus* in KCl buffer occurred at pH of 10 and the velocity was approximately 22 µm per second. These data suggest that K^+^-supported motility of *B. alcalophilus* is similar in speed or slightly faster than Na^+^-supported motility. We then further probed the cation preference for swimming by *B. alcalophilus* in comparison with *B. pseudofirmus* OF4 under conditions in which different Na^+^/K^+^ ratios were compared with Na^+^ and K^+^ addition alone in the assay media. The swimming speeds of *B. alcalophilus* and *B. pseudofirmus* OF4 were measured at pH 9.0 in liquid buffer that contained 200 mM NaCl, 150 mM NaCl plus 50 mM KCl, 100 mM NaCl plus 100 mM KCl, 50 mM NaCl plus 150 mM KCl, or 200 mM KCl (Figure 4C). The velocity of *B. pseudofirmus* OF4 was clearly dependent upon the Na^+^ concentration, and no swimming was observed in the absence of Na^+^. By contrast, the velocity of *B. alcalophilus* was modestly decreased under elevated Na^+^ and reduced K^+^ conditions.

**Figure 5:**
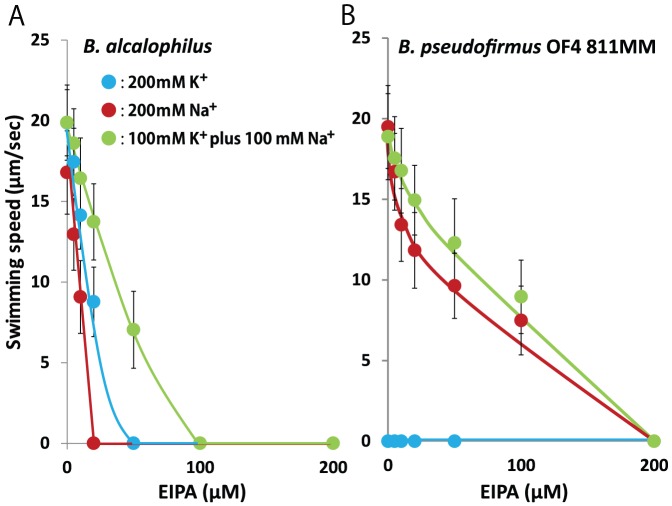
Effect of the Na^+^ channel inhibitor EIPA on motility. The red line and red filled circles show the data for the *B. pseudofirmus* strain, and the blue line and blue filled circles show the data for the *B. alcalophilus* strain in Tris-HCl buffer that contained 5 mM glucose and 200 mM NaCl (pH 9.0) *(A)* or 200 mM KCl (pH 9.0) *(B)*. The swimming speed was determined as described in the Materials and Methods. The results that are shown represent the averages of three independent experiments in each of which the swimming speeds of 20 independent cells as calculated in each experiment. The error bars indicate the standard deviations of the values.

Finally, the effects of the addition of the Na^+^ channel inhibitor EIPA on the swimming speed of the strains were examined (Figure 5). The swimming speed of *B. alcalophilus* was sensitive to EIPA in both Na^+^ and K^+^ buffer. We previously suggested that EIPA binding sites are present in both the MotP and MotS subunits and that this inhibitor blocks K^+^ passage [5].

### A Single Mutation in BA-MotS Change the Profile of the Bi-functional BA-MotPS Stator to a Sodium-only Coupled Stator Profile

**Figure 6:**
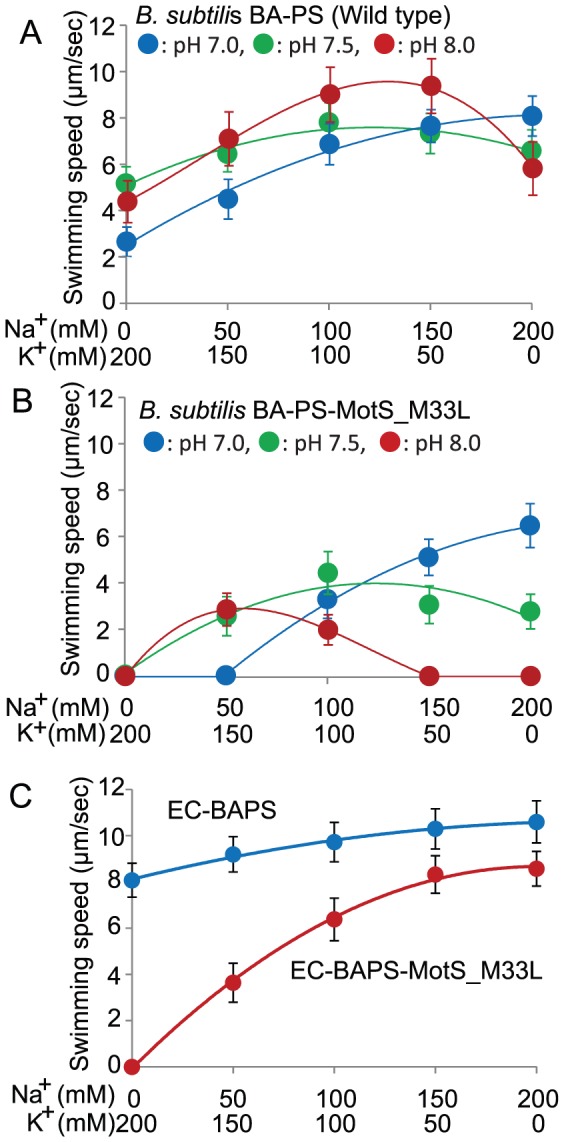
The effect of KCl and NaCl on swimming speed of *B. subtilis* and *E.coli* strains. The effect of KCl and NaCl on swimming speed of *B. subtilis* strains *(A)* and *(B)*, The velocity was measured at several different pH values in phosphate buffer that contained 200 mM Na^+^, 150 mM Na^+^ plus 50 mM K^+^, 100 mM Na^+^ plus 100 mM K^+^, 50 mM Na^+^ plus 150 mM K^+^, or 200 mM K^+^. The blue line and blue filled circles, the green line and green filled circles, and the red line and red filled circles show the data at pH 7.0, 7.5, and 8.0, respectively. The effect of KCl and NaCl on swimming speed of *E. coli* strain *(C)* The velocity was measured at pH 7.0 in phosphate buffer that contained 200 mM Na^+^, 150 mM Na^+^ plus 50 mM K^+^, 100 mM Na^+^ plus 100 mM K^+^, 50 mM Na^+^ plus 150 mM K^+^, or 200 mM K^+^. The blue line and blue filled circles and the red line and red filled circles show the data for EC-BAPS and EC-BAPS-MotS_M33L, respectively. The swimming speed was determined as described in the Materials and Methods section. The results that are shown represent the averages of three independent experiments in each of which the swimming speeds of 20 independent cells as calculated in each experiment. The error bars indicate the standard deviations of the values.

The MotB-like proteins are major determinants of the ion-specificity of the *B. subtilis* MotAB *vs.* MotPS systems [13], [14]. The next challenge was to change the apparent dual specificity of the *B. alcalophilus* MotPS system to one that primarily uses sodium or potassium. We hypothesized that replacing the non-consensus BA-MotS-M33 located in the single transmembrane segment with the conserved leucine among sodium type stators might lead to motility dependent only on sodium (Figure 1). To directly compare the swimming properties conferred by BA-MotPS (wild type) with those conferred by BA-MotPS-MotS_M33L, the two flagella-encoding loci, BA-*motPS* and BA-*motPS*-MotS_M33L, were introduced into the *amyE* chromosomal locus of a *B. subtilis* strain (designated ΔABΔPS) from which both native BS-*motAB* (*motAB* of *B. subtilis*) and BS-*motPS* (*motPS* of *B. subtilis*) were deleted. The resulting *B. subtilis* mutant strains expressing BA-*motPS* or BA-*motPS*-MotS_M33L were named BA-PS and BA-PS-MotS_M33L, respectively. Each gene pair was under control of the *B. subtilis motAB* promoter. Western blot analyses confirmed the presence of a protein band corresponding to BA-MotP in these strains (Figure. S2). Both stators restored motility to the non-motile ΔABΔPS strain on soft agar plates. We next determined which cations are preferred for flagellar rotation in these strains (Figure 6). The swimming speeds of BA-PS and BA-PS-MotS_M33L were measured at several pH values in phosphate buffer that contained 200 mM NaCl, 150 mM NaCl plus 50 mM KCl, 100 mM NaCl plus 100 mM KCl, 50 mM NaCl plus 150 mM KCl, or 200 mM KCl. We used phosphate buffer instead of Tris buffer for this swimming assay because the motility of *B. subtilis* is significantly inhibited by Tris buffer. The swimming of BA-PS was observed in the absence of Na^+^ at all pH values; maximum swimming speed was observed at pH 7.5 and the velocity was approximately 5 µm per second (Figure 6A). The velocity at pH 7 was increased when the Na^+^ concentration was increased up to 200 mM. The velocity at pH 7.5 and 8 was also stimulated by up to 100 mM and 150 mM Na^+^ concentrations, respectively. At all pH values, *B. subtilis* BA-PS, which expressed the native *B. alcalophilus* stator, exhibited a significant capacity to swim without added sodium but also exhibited stimulation by sodium. The results suggested that the BA-PS rotor retained its original ion-coupling capacity in the heterologous neutralophilic host. No swimming was observed by *B. subtilis* BA-PS-MotS_M33L in the absence of Na^+^ at any of the pH values (Figure 6B). At pH 7, swimming of BA-PS-MotS_M33L was clearly dependent upon added sodium ion and required a threshold Na^+^ concentration of 50 mM. At pH 7.5, stimulation by added sodium was modest. At pH 8, swimming supported by BA-PS-M33L in the neutralophile was stimulated up to 50 mM Na^+^ and then no swimming was observed above 150 mM Na^+^ concentrations. The results were consistent with the observations in the native host suggesting that the BA-PS-MotS_M33L stator lost its original potassium-coupling capacity.

### Both the *motPS* gene and *motPS*-MotS_M33L gene of *B. alcalophilus* complement motility-deficient *E. coli* RP6894

A stator-defective mutant, *E. coli* RP6894 was used to expressed the *B. alcalophilus motPS* genes from a plasmid, pBAPS (generating a transformant designated EC-BAPS) or to express the *B. alcalophilus motPS*-MotS_M33L genes from plasmid pBAPS-MotS_M33L (generating a strain designated EC-BAPS-MotS_M33L). The *E. coli* mutant transformants exhibited bands corresponding to BA-MotS in Western analyses (Figure S2). Both of these *E. coli* transformants exhibited motility, so the swimming velocity of EC-BAPS and EC-BAPS-MotS_M33L was measured at pH 7 in phosphate buffers that contained 200 mM Na^+^, 150 mM Na^+^ plus 50 mM K^+^, 100 mM Na^+^ plus 100 mM K^+^, 50 mM Na^+^ plus 150 mM K^+^, or 200 mM K^+^. The velocity of EC-BAPS-MotS_M33L was clearly dependent upon the Na^+^ concentration, and no swimming was observed in the absence of Na^+^ (Figure 6C). The velocity of EC-BAPS was not dependent upon added Na^+^ in the presence of K^+^ but it was slightly increased with increasing Na^+^ concentrations in the buffer. The results suggested MotS and MotS_M33L is retaining their distinct ion selectivities when functioning in combination with MotP to power a flagellar motor in *E. coli*.

### Complementation studies in K^+^-uptake-deficient *Escherichia coli* mutant strains carrying the *motPS* gene and the *motPS*-MotS_M33L gene of *B. alcalophilus*

**Figure 7:**
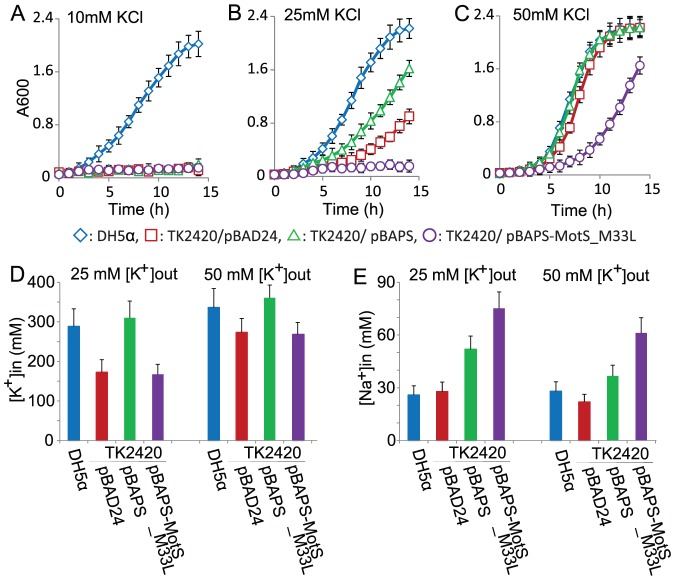
Effect of KCl on the growth and intracellular ion content of various *E. coli* TK2420 transformants. The growth of *E. coli* strain DH5αMCR transformed with control plasmid pBAD24 (filled blue circles) and *E. coli* strain TK2420 transformed with pBAD24 (open blue circles), pBAPS (filled red circles) and pBAPS-MotS_M33L (open red circles). Cells were shaken in the TK2420 minimal medium adding 10 mM *(A)*, 25 mM *(B)* or 50 mM *(C)* KCl at 37°C under aerobic conditions. Cell growth was monitored at 600 nm. Intracellular [K^+^] and [Na^+^] levels in *E. coli* DH5αMCR transformed with control plasmid pBAD24 (filled light blue bar) and *E. coli* strain TK2420 transformed with pBAD24 (open light blue filled bar), pBAPS (filled red bar) and pBAPS-MotS_M33L (open red bar). Cells were shaken in the TK2420 minimal medium adding 25 mM *(D)*, or 50 mM *(E)* KCl at 37°C under aerobic conditions. The results are the averages of three independent duplicate experiments, with error bars representing the standard deviations.

While the profiles of cation dependence of *B. alcalophilus* MotPS strongly suggested a capacity of the stator to couple motility to K^+^, the profiles did not provide direct evidence for actual inward translocation of K^+^ that was dependent upon MotS. We hypothesized that if the stator protein MotPS from *B. alcalophilus* is incorporated in a functional flagellar motor and takes up K^+^ during rotation, this K^+^ uptake would complement the growth defect of K^+^ uptake-deficient *E. coli* TK2420. That is, the growth of the cells at growth-limiting K^+^ concentrations would be better in transformants expressing a functional K^+^-responsive stator than in the *E. coli* TK2420 transformed with an empty vector (pBAD24). Further, since the cytotoxicity of Na^+^ is exacerbated by sub-optimal cytoplasmic concentrations of K^+^ [25], a transformant expressing the BA-PS-MotS_M33L stator might exhibit even less growth than the empty vector control. As shown in Figure 7, *E. coli* TK2420 transformed with the empty vector requires >10 mM added K^+^ for growth whereas an *E. coli* DH5α (pBAD24) control, with no deficit in K^+^ uptake, (Figure 7A, B) grows only a little sub-optimally at 10 mM KCl relative to its growth at higher concentrations. By contrast, *E. coli* TK2420 carrying empty vector (pBAD24), pBAPS and pBAPS-MotS_M33L were all unable to grow in minimal medium with 10 mM K^+^. In the minimal medium with 25 mM KCl added, the doubling times of *E. coli* DH5α (pBAD24), and strainTK2420 carrying empty vector (pBAD24) or pBAPS were approximately 1.5, 3.2 or 2.7 h, respectively (Figure 7B). On the other hand, the growth of *E. coli* TK2420 harboring pBAPS-MotS_M33L was not complemented (Figure 7B). The growth of *E. coli* TK2420 transformed with either empty vector or pBAPS was enhanced when 50 mM KCl was added to the medium. The two transformants respectively exhibited doubling times of approximately 1.4 or 1.3 h in the presence of added KCl (Figure 7C); these doubling times were comparable to the growth of the DH5α (pBAD24) control. By contrast, the *E. coli* TK2420 transformant with pBAPS-MotS_M33L exhibited a long lag and a doubling time of 2.1 h.

The wild type *E. coli* (DH5aMCR) clearly grew better than the mutant TK2420 strain carrying the vector alone at sub-optimal [K^+^]. The osmolality of minimal medium with 25 mM K^+^ is about 0.2 osmolar. The intracellular K^+^ content of *E. coli* cells grown under these conditions had been found to be about 220 mmol K^+^ per liter of cytoplasmic H_2_O [21]. The concentration obtained for the wild type in this study (289 mM) is 1.3 times higher than this previously published value but is still in reasonably good agreement with it. The intracellular K^+^ content of *E. coli* TK2420 carrying empty vector (pBAD24), pBAPS and pBAPS-MotS_M33L grown in minimal medium with 25 mM K^+^ was 60%, 107%, and 58% of that of the wild type, respectively (Figure 7D). On the other hand, the intracellular Na^+^ content of TK2420 carrying empty vector (pBAD24), pBAPS and pBAPS-MotS_M33L grown in minimal medium with 25 mM K^+^ was 107%, 200%, and 290% of that of the wild type, respectively (Figure 7D). A higher K^+^ content is presumably why the generation times for *E. coli* TK2420 carrying pBAPS are better than the TK2420 strain transformed with empty vector. Poor growth of *E. coli* TK2420 carrying pBAPS-MotS_M33L at 25 mM K^+^ and growth inhibition at 50 mM K^+^ presumably result from the combination of reduced K^+^ uptake capacity at 25 mM K^+^ and uptake of cytotoxic Na^+^ at both 25 and 50 mM K^+^ (Figure 7D, E). The results suggest that BA-MotPS supports actual K^+^ uptake, with a role as a coupling ion, while the the Na^+^ uptake catalyzed BA-MotPS-MotS_M33L is consistent with the earlier data that it retains this uptake capacity while having lost a capacity for K^+^ uptake.

## Discussion

**Figure 8:**
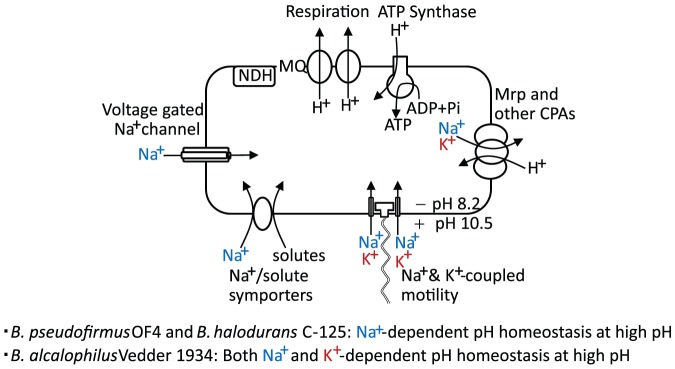
The Na^+^ and K^+^ cycles of alkaliphiles. A diagrammatic illustration of the pH homeostasis capacity of the Na^+^- and Na^+^ plus K^+^-dependent alkaliphiles and elements of their membrane-associated Na^+^, K^+^ and H^+^ translocation pathways.

A major finding of this study is that *Bacillus alcalophilus* Vedder 1934, an alkaliphile strain that was originally isolated from human feces, differs from the most intensively characterized alkaliphilic *Bacillus* strains, such as *B. pseudofirmus* OF4 and *Bacillus halodurans* C-125 in being able to support both cytoplasmic pH homeostasis and flagellar motility at high pH using K^+^ when the Na^+^ concentrations are low. In addition, it raises interesting questions about the nature of the potassium cycle that supports pH homeostasis, when Na^+^ is absent or insufficient. This potassium cycle is expected to differ from the strictly Na^+^-dependent cycle characterized for both *B. pseudofirmus* OF4 and *B. halodurans* C-125. In those two Na^+^-dependent alkaliphiles, cytoplasmic pH homeostasis at high pH, depends upon use of a hetero-oligomeric Na^+^/H^+^ antiporter, the Mrp (or Cation/Proton Antiporter-3 family) antiporter to catalyze net uptake or H^+^ in exchange cytoplasmic Na^+^ during aerobic growth on malate (Figure 8) [26], [27]. The antiporters catalyze electrogenic efflux of cytoplasmic Na^+^ in exchange for a greater number of H^+^, so that the transmembrane electrical potential (negative inside relative to outside), ΔΨ, can energize the antiport. Coupling of motility, uptake of a large number of solutes and use of a voltage-gated NaChBac type sodium channel all contribute to sufficient return of Na^+^ to the cytoplasm to ensure ongoing antiport activity in support of pH homeostasis (Figure 8) [26], [28], [29]. Earlier work of Krulwich and colleagues revealed that alkaliphilic *B. alcalophilus* ATCC27647, which originated from the Vedder 1934 strain, uses electrogenic Na^+^/H^+^ antiporter activity and Na^+^/solute symport to facilitate growth at alkaline pH [30], [31]. However, it is clear from the current findings that a parallel but distinct cycle of potassium is present in *B. alcalophilus*.

Although Na^+^ is usually required for, or greatly enhances growth of alkaliphilic bacteria, some alkaliphiles have long been known to grow in alkaline media that contain K_2_CO_3_ but lack Na_2_CO_3_ [32], [33]. In addition, in caterpillars, whose gut is known to constitute an alkaline environment, the elevation of gut pH is linked to potassium transport and net accumulation of K_2_CO_3_ [34] and termite-derived alkaliphiles show NaCl sensitivity and preference of K^+^ over Na^+^ during growth at high pH [35]. While the membranes of some bacteria may be too leaky to K^+^ to rely on K^+^-coupled transporters and/or pumps to carry out physiologically important energetic work [36], there are precedents for such usage of potassium in both eukaryotes and in non-alkaliphilic as well as alkaliphilic prokaryotes. K^+^/H^+^ antiporters have been reported in both prokaryotes and eukaryotes [37], [38], and potassium has been shown to be a counter-ion for a sodium-proton-glutamate transporter in eukaryotes [39] and for a sodium/proton-potassium antiporter in a non-alkaliphilic *Bacillus* [40]. Potassium also serves as coupling ion for the KAAT1 potassium/amino acid transporter in the larval insect mid-gut [41] and K^+^ can also couple to leucine transport in brush border membrane vesicles from *Manduca sexta*, a well-studied insect model [42]. When *B. alcalophilus* uses K^+^ for cytoplasmic pH homeostasis, it is likely that an electrogenic K^+^/H^+^ antiporter is employed. It will be of interest to determine whether a K^+^-translocating multi-subunit Mrp antiporter is of major importance in *B. alcalophilus*, since Na^+^-translocating Mrp antiporters have been shown to play critical roles in other alkaliphiles that depend completely upon Na^+^ for pH homeostasis [26], [27]. K^+^-translocating Mrp type antiporters have been described in other bacteria [38], [43], [44] but not yet characterized in an alkaliphilic *Bacillus*. It remains possible that an electrogenic antiporter type other than Mrp has a major role in K^+^-dependent pH homeostasis in this setting (Figure 8). An electroneutral K^+^/H^+^ antiporter was detected in *B. alcalophilus* [31], but information about electrogenic K^+^/H^+^ antiporters is still lacking for this organism. It will be of further interest to ascertain whether there are novel channels or symporters that replenish cytoplasmic potassium and whether *B. alcalophilus* utilizes, in part, the K^+^ gradient to augment the ΔΨ as long at the K^+^ is outwardly directed.

The other major finding in this study, and the most unprecedented one, is the evidence of potassium-coupling to flagellar rotation. The finding that the potassium uptake-defective *E. coli* strain TK2420 is complemented by the wild-type *B. alcalophilus* BA-MotPS but not by the mutant BA-MotPS_M33L (Figure 7) supports the conclusion that the K^+^ is playing the role of coupling ion and being translocated into the cell during flagellar rotation. The consequences of mutating the MotB residue that is uniquely methionine in *B. alcalophilus* is yet another example verifying the importance of the residue at this particular position for the ion-coupling properties. We note that the *B. pseudofirmus* OF4 stator tends, in general, to be inhibited more than that of *B. alcalophilus* at near neutral pH. This inhibition has been attributed to strong competitive inhibition by protons on the Na^+^-coupled stator of this strain [45], which is also observed with the Na^+^-coupled symporters of *B. pseudofirmus* OF4 at pH 7.5 [46]. This putative inhibitory effect, which is less pronounced or absent in the experiments with *B. alcalophilus*, has been interpreted as one of several indications of the “hard wiring” of *B. pseudofirmus* OF4 for growth at high pH at the expense of optimized growth at near neutral pH [47]. It is felicitous that the BA-MotPS is functional and retains its native coupling properties in an *E. coli* strain because this opens up the opportunity for future experiments using single molecular microscopic techniques of analyzing the flagellar motor that are already established in *E. coli* [48]. Further work may also make it possible to find a mutant form of the stator that lacks or has severely diminished capacity for Na^+^-coupling as was possible to achieve with the dual function stator of *B. clausii* [14].

### Conclusions

A major finding of the current study is the demonstration that a single bacterial flagellar stator of *B. alcalophilus* can couple motility to either sodium or potassium ions. This finding may facilitate the identification of additional examples of other cation coupling capacities among the rapidly growing number of bacteria that exhibit natural motility and for which the genomic sequences are available. Further mutagenesis studies and, ultimately, the correlation of these types of data with high-resolution structural data that are related to these and other transporters that catalyse both sodium- and potassium-coupled bioenergetics will facilitate the generation of detailed models of the cation-binding sites, including the basis for competitive proton binding at near neutral pH.

## Supporting Information

Figure S1 **Stained flagellar of** ***B. alcalophilus*** **.** Cells were stained with staining solution that contained 5% (wt/vol) tannic acid as described in Materials and Methods.
(EPS)

Figure S2 **Western blot detection of MotP proteins from** ***B. alcalophilus*** **expressed in** ***B. subtilis*** **and** ***E. coli*** **transformants.** Three *B. subtilis* strains, (A) ΔABΔPS, BA-PS and BA-PS-MotS-M33L, and six *E. coli* strains, (B) RP6894 carrying pBAD24, pBAPS, or pBAPS-MotS-M33L, and (C) TK2420 carrying pBAD24, pBAPS, or pBAPS-MotS-M33L, were grown as described in the Materials and Methods section. Each sample was subjected to SDS-PAGE followed by immunoblotting with anti-MotP antibody. The arrowhead on right-hand side of each panel indicates the MotP protein. The asterisks on right-hand side of the panel A indicate the non-specific bands.
(EPS)
